# Clinical effects of *Emblica officinalis* fruit consumption on cardiovascular disease risk factors: a systematic review and meta-analysis

**DOI:** 10.1186/s12906-023-03997-8

**Published:** 2023-06-09

**Authors:** Paul D. S. Brown, Nicole Ketter, Mathew Vis-Dunbar, Brodie M. Sakakibara

**Affiliations:** 1 Southern Medical Program, Faculty of Medicine, University of British Columbia Okanagan Campus, 1088 Discovery Avenue, Kelowna, BC V1V 1V7 Canada; 2 Department of Biology, Irving K. Barber Faculty of Science, University of British Columbia Okanagan Campus, 3187 University Way, ASC 413, Kelowna, BC V1V 1V7 Canada; 3 Centre for Chronic Disease Prevention and Management, University of British Columbia Okanagan Campus, 1088 Discovery Avenue, Kelowna, BC V1V 1V7 Canada; 4 Department of Occupational Science and Occupational Therapy, University of British Columbia, T325 - 2211 , Wesbrook Mall, Vancouver, BC V6T 2B5 Canada

**Keywords:** Meta-analysis, Cardiovascular disease, Cholesterol, Inflammation, *Emblica officinalis*, Chronic disease, Lipids, Amla, *Phyllanthus emblica*, Atherosclerosis

## Abstract

**Background:**

*Emblica officinalis* (EO) fruit consumption has been found to have a beneficial effect on cardiovascular disease (CVD) physiological risk factors in preliminary clinical intervention trials; however, questions remain regarding the overall effectiveness of EO on CVD risk. The purpose of this systematic review and meta-analysis is to: 1) systematically describe the clinical research examining EO; and 2) quantitatively assess the effects of EO on CVD physiological risk factors.

**Methods:**

The Pubmed, Embase, Web of Science, and Google Scholar electronic platforms were searched for relevant randomized controlled trials (RCTs) published until April 7, 2021. Studies were included if they involved adults (age ≥ 18 years) ingesting a form of EO fruit; included blood lipids, blood pressure, and/or inflammatory biomarkers as outcomes; had clearly defined intervention and control treatments with pre- and post-intervention data; were peer-reviewed; and were written in English. Studies were excluded if they compared EO with another risk reduction intervention without a usual care control group. RCTs were assessed for methodological quality using the Cochrane risk-of-bias version 2 (ROB2) tool, qualitatively described, and quantitatively evaluated using random and fixed effect meta-analysis models.

**Results:**

A total of nine RCTs (*n* = 535 participants) were included for review. Included studies followed parallel-group (*n* = 6) and crossover (*n* = 3) designs, with EO dosage ranging from 500 mg/day to 1500 mg/day, and treatment duration ranging from 14 to 84 days. Meta-analyses revealed EO to have a significant composite effect at lowering low-density lipoprotein cholesterol (LDL-C; Mean difference (MD) = -15.08 mg/dL [95% Confidence interval (CI) = -25.43 to -4.73], I^2^ = 77%, prediction interval = -48.29 to 18.13), very low-density lipoprotein cholesterol (VLDL-C; MD = -5.43 mg/dL [95% CI = -8.37 to -2.49], I^2^ = 44%), triglycerides (TG; MD = -22.35 mg/dL [95% CI = -39.71 to -4.99], I^2^ = 62%, prediction interval = -73.47 to 28.77), and high-sensitivity C-reactive protein (hsCRP; MD = -1.70 mg/L [95% CI = -2.06 to -1.33], I^2^ = 0%) compared with placebo.

**Conclusions:**

Due to statistical and clinical heterogeneity in the limited number of clinical trials to date, the promising effects of EO on physiologic CVD risk factors in this review should be interpreted with caution. Further research is needed to determine if EO offers an efficacious option for primary or secondary prevention of CVD as either monotherapy or adjunct to evidence-based dietary patterns and/or standard pharmacotherapy.

**Supplementary Information:**

The online version contains supplementary material available at 10.1186/s12906-023-03997-8.

## Background

Cardiovascular disease (CVD) is the leading cause of death globally, accounting for ~ 17.8 million deaths annually [1]. Mortality associated with CVD is expected to increase to > 22.2 million per year by 2030 [2]. Due to increasing prevalence, further efforts are required for both primary and secondary prevention of CVD. Aging demographics combined with improved survival post-cardiovascular event contribute to the growing pool of individuals living with established CVD [3]. Secondary prevention of subsequent events via improvement in modifiable CVD risk factors can help reduce morbidity and mortality in this growing population [3].

Modifiable risk factors associated with CVD are both behavioural and physiological. Research indicates a linear progression of risk factors leading to CVD, beginning with unhealthy lifestyle behaviours (e.g., physical inactivity, poor nutritional intake), leading to uncontrolled physiological risk factors, ultimately translating to CVD. Dyslipidemia, inflammation, and hypertension are common physiological risk factors for developing CVD via the progression of atherosclerosis [1]. Evidence-based dietary patterns have been developed to improve CVD physiological risk factors, including the Dietary Approaches to Stop Hypertension (DASH) diet [4] and the Portfolio diet [5, 6]. Although these dietary interventions have been associated with improvement in physiological CVD risk factors, CVD remains a significant global health concern. Therefore, the identification of efficacious, safe, affordable, and convenient options for primary or secondary prevention of CVD as either monotherapy or adjunct to evidence-based dietary patterns and/or standard pharmacotherapy is essential [7]. Furthermore, metabolic syndrome is a multicomponent risk factor for CVD and type 2 diabetes mellitus (T2DM) [1]. Metabolic syndrome is diagnosed when any three or more of the following five cardiometabolic risk factors are present: 1) hypertriglyceridemia, 2) decreased high-density lipoprotein cholesterol (HDL-C), 3) hypertension, 4) hyperglycemia, or 5) central adiposity [1]. Metabolic syndrome increases the risk of CVD mortality and all-cause mortality even for those with metabolic syndrome without T2DM [8]. Therefore, a single agent with the ability to produce beneficial changes in multiple cardiometabolic risk factors would be ideal when treating patients living with metabolic syndrome.

*Emblica officinalis* (EO)—also known as *Phyllanthus emblica*, Indian gooseberry in English, Amla in Hindi, and Amalaki in Sanskrit [9]—is a 5-25 m tall deciduous tree, native to tropical and subtropical regions of India, Nepal, Sri Lanka, and throughout South-East Asia to southern China [10]. Although many components of the EO plant (e.g., root bark, stem bark, leaves) are traditionally used in Ayurveda, an Indian indigenous system of medicine, the edible fruit is typically used the most for health reasons [7]. EO berries are spherical and smooth, growing to 2-5 cm in diameter [11]. EO berries are initially pale green in colour, changing to yellow when mature [10]. EO fruit, and formulations incorporating EO fruit, have traditionally been used as dietary supplements to treat an abundance of health ailments, including fever, jaundice, anemia, cough, asthma, headache, dyspepsia, ophthalmic disorders, vomiting, leprosy, diabetes, and menorrhagia [9].

The phytoconstituents of EO fruit include many bioactive compounds including hydrolysable tannins (e.g., chebulinic acid, chebulagic acid, corilagin, punigluconin, pedunculagin, emblicanin A and B), alkaloids, phenols (e.g., gallic acid, ellagic acid, pyrogallol), amino acids, carbohydrates (e.g., pectin), vitamins (e.g., ascorbic acid), flavonoids (e.g., quercetin, kaempferol, rutin), and organic acids (e.g., citric acid) [12]. EO fruit is a rich source of ascorbic acid (vitamin C), with 470-680 mg per 100 g [9]. Vitamin C accounts for ~ 45–70% of the total antioxidant activity of the EO fruit, along with tannins (particularly punigluconin, pedunculagin, emblicanin A and B), flavonoids, and ellagic acid [11]. Furthermore, experimental research indicates the EO fruit to have antibacterial [13], antidiabetic [14], antidiarrheal [15], antihyperlipidemic [16, 17], antioxidant [18], antipyretic [19], anti-hyperthyroid [20], antitussive [21], antiulcer [22], chemopreventive [23], cognitive enhancing [24], gastroprotective [25], hepatoprotective [26], nephroprotective [27], skin antiaging [28], and wound healing [29] properties, among many others.

Preliminary clinical interventional trials have also shown promising results of EO fruit consumption on a variety of health conditions, including cardiovascular disease [9, 12, 30, 31]. Specifically, significant improvements in participant blood lipids and/or biomarkers of inflammation following consumption of EO fruit in various forms [32–49]. These initial studies have subsequently led to randomized controlled trials (RCTs) investigating the effects of EO on CVD physiological risk factors [50–58]. Thus, a body of evidence now exists on the effects of EO on physiological risk, however, these effects have not been systematically reviewed or meta-analyzed. The purpose of this systematic review and meta-analysis is to: 1) systematically describe the clinical research examining EO; and 2) quantitatively assess the effects of EO on CVD physiological risk factors, including blood lipids, blood pressure, and biomarkers of inflammation.

## Methods

This systematic review and meta-analysis protocol was not registered; however, the reporting in this review follows the Preferred Reporting Items for Systematic Reviews and Meta-Analyses (PRIMSA) guidelines [59].

### Inclusion/exclusion criteria

Randomized controlled trials were included for review if they involved adults (age ≥ 18 years), with any diagnostic condition, ingesting a form of the EO fruit (no polyherbal formulations); physiological CVD risk factor outcomes used in clinical practice (e.g. blood lipids, blood pressure, and/or inflammatory biomarkers); had clear definitions of intervention and placebo control treatments, such as proprietary extract descriptions and placebo constituents; had pre- and post-intervention data; were published in a peer-reviewed journal; and were written in English. Studies were excluded if they compared EO with another risk reduction intervention without a usual care control group. Cross-over trials were considered appropriate and included in this review due to the temporary effect of EO consumption and the stability of the patient population’s health status [60].

### Information sources/Search

The Pubmed, Embase, Web of Science, and Google Scholar electronic platforms were searched for relevant literature published up until April 7, 2021 using the search strategy detailed in Appendix A that was collaboratively developed with the university librarian. The Cochrane Database of Systematic Reviews in addition to the electronic platforms were searched for relevant reviews on EO. The reference lists of all relevant papers and reviews were searched for additional studies.

### Study selection

All results from the electronic search were imported into a systematic review management program [61]. After duplicates were removed, the title and abstract screening was performed by two study authors independently. If both authors deemed an abstract relevant, it moved on to the full text review; discrepancies in judgement were resolved via discussion and engagement of another reviewer. Full texts of relevant studies were read by two authors independently. Any discrepancy was discussed to determine final eligibility. Additional papers of interest found in reference lists were obtained and read to determine eligibility.

### Data collection process

Data from relevant studies were extracted by the second author and tabulated for comparison. Extracted data included author(s), year, country, study design, sample size, participant characteristics (age, sex, medical diagnoses and medication information relevant to each study’s inclusion criteria, and anthropometric and physiological data), details of the intervention and control treatments, outcome measures, and key results. Outcome measures included blood lipids (total cholesterol [TC], triglycerides [TG], LDL-C, HDL-C, and very low-density lipoprotein cholesterol [VLDL-C]), blood pressure (systolic and diastolic), and inflammatory biomarkers (high-sensitivity C-reactive protein [hsCRP]).

### Study risk of bias assessment

Methodological quality of each study was completed using version 2 of the Cochrane risk-of-bias tool (ROB2) [62]. The ROB2 tool consists of 5 domains for parallel studies and 6 domains for cross-over studies. These domains include risk of bias arising from the randomization process, due to deviations from intended interventions, due to missing outcome data, in measurement of the outcome, and selection of the reported result. The cross-over tool also includes risk of bias arising from period and carryover effects. For each domain, there is a series of signaling questions and response options include “yes”, “probably yes”, “probably no”, “no”, and “no information”. Each domain is then given a risk of bias judgement of low, some concern or high using a pre-determined algorithm.

### Meta-analyses

Effect sizes were calculated for all outcome measures regardless of dosage. Study data were meta-analyzed using the mean difference (MD) for continuous data. The MD is used as a summary statistic to measure the absolute difference between the mean value in two groups when the outcome measures are made on the same scale [63]. If standard error of the mean was reported, it was converted to standard deviation by multiplying by the square root of the sample size [64]. Forest plots were used to visually display mean differences in outcomes between treatment and control groups for each study. The cross-over trials were analyzed as parallel trials when paired-analyses data and first period only data were not reported, which was the case for all cross-over trials [65].

The main analysis estimates the effect size of all interventions, regardless of dosage of EO on physiological risk factors. In the case where studies had two intervention groups, one receiving 500 mg/day and one receiving 1000 mg/day, the two intervention groups were combined as recommended in the Cochrane handbook [66]. This formula can be found in Appendix B. The meta-analyses estimated the pre-post effects immediately following the completion of the intervention. The I^2^ statistic was used to determine statistical heterogeneity. A random effects model was used if the I^2^ value was greater than 50%, signifying notable heterogeneity [67], otherwise a fixed effect model was used. Prediction intervals were reported for all random effects models to identify the range of true effect sizes and were calculated using a spreadsheet provided in Borenstein et al. [68]. All analyses were performed using RevMan 5.4 [69], at an alpha set at 0.05.

## Results

**Fig. 1:**
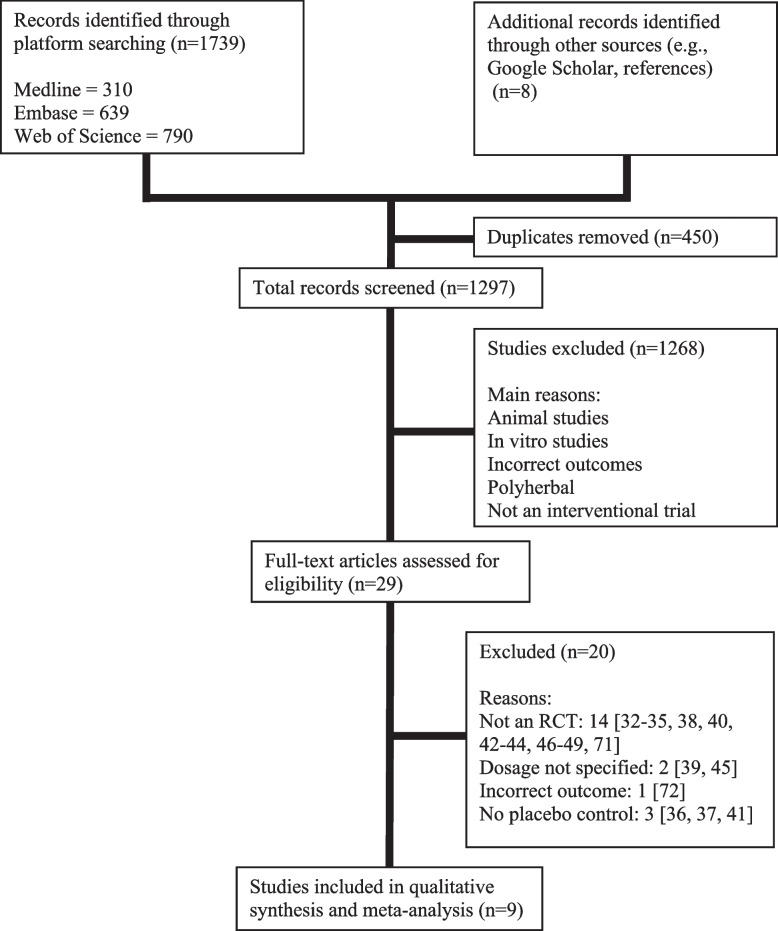
Selection process of studies examining the effects of EO fruit consumption on CVD risk factors [71, 72]. *EO Emblica officinalis*, *CVD* Cardiovascular disease, *RCT* Randomized controlled trial

The search yielded 310 results from Medline, 639 from Embase, and 790 from Web of Science. The PRISMA flow diagram is shown in Fig. 1. Only the first 200 results from Google Scholar were screened for any additional records as recommended by Bramer and colleagues [70]. After 450 duplicates were removed, the remaining 1297 abstracts were screened. Twenty-nine abstracts remained and their full texts were then assessed for eligibility. Nine studies were included in the descriptive synthesis and quantitative meta-analysis.

Study characteristics of the nine RCTs included for review are presented in Table 1. Overall, the sample sizes ranged between 12 [51, 58] and 150 [54] participants. Duration of treatment ranged between 14 [51, 58] and 84 [54–57] days. Seven of the RCTs were from India [50, 51, 54–58], one from Japan [53], and one from Iran [52]. Eight RCTs were double-blinded (i.e., participants and researcher) [50, 51, 53–58] and one RCT was triple-blinded (i.e., participants, researcher, and data analyzer) [52]. Six RCTs were of parallel-group design [50, 52, 54–57], while the remaining three were of crossover design [51, 53, 58]. All nine RCTs recruited adults (age range 20–74 years). Participant recruitment for each RCT were healthy males [51, 58]; healthy males and females [53]; male smokers [50]; males and females with dyslipidemia [55], T2DM [56], metabolic syndrome [57], uncontrolled hypertension [52], and essential hypertension [54].

**Table 1:** Study characteristics

**Author(s), year** **Study Design** **Country** **ROB2** [62]** Score**	**Participants**	**Intervention/Control Groups**	**Outcome Measures**	**Key Results**
**Biswas et al. 2014** [50] Randomized, double-blind placebo-controlled pilot study Kolkata, India High Risk of Bias	**Population**: Adult smokers (*n* = 30 total) **Sex:** 30 Males **Age**: 20–60 years **Characteristics**: chronic cough, poor immune status, compromised cardiovascular status, and lipid profile	**Intervention**: *n* = 20, 250 mg aqueous EO fruit extract (standardized to contain ≥ 60% w/w low molecular weight hydrolysable tannins) capsule b.i.d. PO for 60 days **Control**: *n* = 10, 250 mg placebo (microcrystalline cellulose, lactose and magnesium stearate) capsule b.i.d. PO for 60 days	TC, LDL-C, HDL-C, TG, hsCRP	Significant decrease in TC, LDL-C, TC/HDL-C ratio, LDL-C/HDL-C ratio, hsCRP, and significant increase in HDL-C after EO compared with baseline
**Fatima et al. 2014** [51] Randomized, double-blind, placebo-controlled crossover study Hyderabad, India Some Concern of Bias	**Population**: Healthy adults (*n* = 12 total) **Sex:** 12 males **Age**: 20–30 years, mean of 25.62 ± 2.32 years **Characteristics**: mean BMI 22.42 ± 2.32 kg/m^2^)	**Intervention**: *n* = 12, 250 mg aqueous EO fruit extract (standardized to contain ≥ 60% w/w low molecular weight hydrolysable tannins) capsule b.i.d. PO for 14 days 14-day washout period **Control**: *n* = 12, placebo capsule contains microcrystalline cellulose (49.7% w/w), lactose (49.5% w/w) and magnesium stearate (0.69% w/w) b.i.d. PO for 14 days	Automated, radial and aortic SBP and DBP using non-invasive software (SphygmoCor; AtCor Medical, Australia)	Significant decrease in cold pressor stress test induced changes on aortic wave reflections after EO compared with baseline and placebo
**Ghaffari et al. 2020** [52] Randomized, triple-blind, placebo-controlled add-on clinical trial Tabriz, Iran Some Concern of Bias	**Population**: Adults with uncontrolled hypertension (*n* = 81 total) **Sex**: 45 males, 36 females **Age**: 35–74 years, mean of 53.64 ± 10.01 years **Characteristics**: BMI < 30, SBP ≥ 140 mm Hg, DBP ≥ 90 mm Hg or both, and SBP ≥ 150 mm Hg for patients older than 60 years), maximum of 2 antihypertensive drugs for at least 8 weeks, a minimum of one month must have elapsed from the start of medication	**Intervention**: *n* = 41, 500 mg EO fruit powder (fresh EO fruit dried then powdered) capsule t.i.d. PO after meal for 8 weeks **Control**: *n* = 40, placebo (wheat starch powder) capsule t.i.d. PO after meal for 8 weeks	Automated, brachial SBP and DBP TC, LDL-C, HDL-C, TG	Significant decrease in SBP and DBP after EO compared with baseline and control group
**Kapoor et al. 2020** [53] Randomized, double-blind, placebo-controlled crossover study Japan Some Concern of Bias	**Population**: Healthy adults (*n* = 13 total) **Sex**: 6 males, 7 females **Age**: 36–67 years, mean of 51.9 ± 2.8 years **Characteristics**: Mean BMI 25.1 ± 0.63 kg/m^2^, with elevated TG, lower HDL-C, and average blood fluidity	**Intervention**: *n* = 13, 125 mg aqueous EO fruit extract powder (EO fruit pulp hydrolysed with pectinase followed by centrifugation with the supernatant spray dried) capsule q.i.d. PO (2 capsules after breakfast + 2 after dinner) for 4 weeks (each capsule contained 125 mg EO + 125 mg dextrin) 3-week washout period **Control**: *n* = 13, 250 mg dextrin capsule q.i.d. PO (2 capsules after breakfast + 2 after dinner every day) for 4 weeks	Automated, brachial SBP and DBP TC, LDL-C, HDL-C, TG	Significant increase in HDL-C after 2 weeks (but not 4 weeks) of EO compared with baseline
**Shanmugarajan et al**. 2021 [54] Randomized, double-blind, placebo-controlled clinical trial Puducherry, India Some Concern of Bias	**Population**: Adults with essential hypertension (*n* = 150 total) **Sex**: 119 males, 31 females **Age**: Intervention: mean of 58.8 ± 8.68 years, control: mean of 60.0 ± 9.42 years **Characteristics**: Essential hypertension on amlodipine 5 mg or enalapril 5 mg and had not yet attained target blood pressure goals (130 mmHg of systolic and 80 mmHg of diastolic blood pressure)	**Intervention**: *n* = 75, 500 mg aqueous EO dried fruit extract powder capsule b.i.d. PO for 12 weeks **Control**: *n* = 75, 500 mg maize starch IP grade capsule b.i.d. PO for 12 weeks	SBP, DBP TC, LDL-C, VLDL-C, HDL-C, TG, hsCRP	NS change in SBP, DBP, TC, LDL-C, VLDL-C, HDL-C, TG, or hsCRP after EO compared with placebo
**Upadya et al. 2019** [55] Randomized, double-blind, placebo-controlled, multicenter clinical trial Southern India Some Concern of Bias	**Population**: Adults with dyslipidemia (*n* = 98 total) **Sex**: 45 males, 53 females **Age**: 30–65 years, intervention: mean of 40.7 ± 10.13 years, control: mean of 42.2 ± 9.20 years **Characteristics**: TG > 200 mg/dL, LDL-C > 130 mg/dL, TC > 200 mg/dL and HDL-C < 40 mg/dL for men and < 50 mg/dL for women Patients were not taking any medication (including herbal product) for management of dyslipidemia in past 4 weeks	**Intervention**: *n* = 49, 500 mg aqueous EO fruit extract powder (fresh, whole EO fruit extracted with ethyl acetate, standardized to contain ≥ 35% polyphenols, 8% triterpenoids, and 10% EO oil) capsule b.i.d. PO (1 capsule after breakfast + 1 after dinner) for 12 weeks **Control**: *n* = 49, 500 mg roasted rice powder capsule b.i.d. PO (1 capsule after breakfast + 1 after dinner) for 12 weeks	TC, LDL-C, VLDL-C, HDL-C, TG, hsCRP	Significant decrease in TC, LDL-C, VLDL-C, and TG after EO compared with placebo
**Usharani et al. 2013** [56] Randomized, double-blind, placebo-controlled study Hyderabad, India Some Concern of Bias	**Population**: Adults with T2DM (*n* = 80 total) **Sex**: 53 males, 27 females **Age**: 30–68 years, intervention 1: mean of 57.60 ± 9.67 years, intervention 2: mean of 57.75 ± 9.86 years, intervention 3: mean of 56.95 ± 8.04 years, control: mean of 56.90 ± 9.17 years **Characteristics**: Fasting blood glucose of 110–126 mg/dL, glycosylated hemoglobin of 7%–9%, on stable antidiabetic medication (metformin 1,500–3,000 mg) for the 8 weeks prior to the screening visit, and endothelial dysfunction	**Intervention 1**: *n* = 20, 250 mg aqueous EO extract (standardized to contain ≥ 60% w/w low molecular weight hydrolysable tannins) capsule b.i.d. PO for 12 weeks **Intervention 2**: *n *= 20, 500 mg aqueous EO extract capsule b.i.d. PO for 12 weeks **Intervention 3**: *n* = 20, 10 mg atorvastatin capsule o.d. PO at bedtime + matching placebo capsule o.d. PO in the morning for 12 weeks **Control**: *n* = 20, placebo capsule b.i.d. PO for 12 weeks. Matching placebo capsules contained microcrystalline cellulose (49.7% w/w), lactose (49.5% w/w), and magnesium stearate (0.69% w/w) as excipients	TC, LDL-C, VLDL-C, HDL-C, TG, hsCRP	Significant decrease in TC, LDL-C, TG, hsCRP and significant increase in HDL-C after both EO dosages and atorvastatin compared with baseline and placebo Significant decrease in VLDL-C after both EO dosages compared with baseline but not placebo Significant decrease in VLDL-C after atorvastatin compared with baseline and placebo
**Usharani et al. 2019** [57] Prospective, randomised, double-blind and placebo-controlled clinical study Hyderabad, India Some Concern of Bias	**Population**: Adults with metabolic syndrome (*n* = 59 total) **Sex**: 43 males, 16 females **Age**: 30–68 years, intervention 1: mean of 57.45 ± 7.44 years, intervention 2: mean of 57.24 ± 8.94 years, control: mean of 56.89 ± 7.39 years **Characteristics**: Endothelial dysfunction	**Intervention 1**: *n* = 20, 250 mg aqueous EO fruit extract (standardized to contain ≥ 60% w/w low molecular weight hydrolysable tannins) capsule b.i.d. PO for 12 weeks **Intervention 2**: n = 21, 500 mg aqueous EO fruit extract capsule b.i.d. PO for 12 weeks **Control**: *n* = 18, placebo (microcrystalline cellulose, lactose and magnesium stearate) capsule b.i.d. PO for 12 weeks	TC, LDL-C, HDL-C, TG, hsCRP	Significant decrease in TC, LDL-C, HDL-C, TG, and hsCRP after both EO dosages compared with baseline and placebo
**Usharani et al. 2017** [58] Randomized, double-blind, placebo-controlled, crossover study Hyderabad, India Some Concern of Bias	**Population**: Healthy adults (n = 12 total) **Sex**: 12 males **Age**: 20–30 years, mean of 24.75 ± 2.01 years **Characteristics**: BMI between 18–24.9 kg/m^2^ and non-smokers	**Intervention**: *n* = 12, 250 mg aqueous EO fruit extract (standardized to contain ≥ 60% w/w low molecular weight hydrolysable tannins) capsule q.i.d. PO (2 capsules b.i.d. PO) for 14 days 14-day washout period **Control**: *n* = 12, 250 mg placebo (microcrystalline cellulose, croscarmellose sodium, silicon dioxide, talc and magnesium stearate) capsule q.i.d. PO (2 capsules b.i.d. PO) for 14 days	Automated, radial and aortic SBP and DBP using non-invasive software (SphygmoCor; AtCor Medical, Australia)	Significant decrease in mental stress test induced changes on aortic wave reflections after EO compared with baseline and placebo

*b.i.d*. Twice daily, *BMI* Body mass index, *DBP* Diastolic blood pressure, *EO Emblica officinalis*, *HDL-C* High-density lipoprotein cholesterol, *hsCRP* High-sensitivity C-reactive protein, *LDL-C* Low-density lipoprotein cholesterol, *o.d.* Once daily, *PO* Orally, *q.i.d.* Four times daily, *SBP* Systolic blood pressure, *t.i.d.* Three times daily, *T2DM* Type 2 diabetes mellitus, *TC* Total cholesterol, *TG* Triglycerides, *VLDL-C* Very low-density lipoprotein cholesterol

**Fig. 2:**
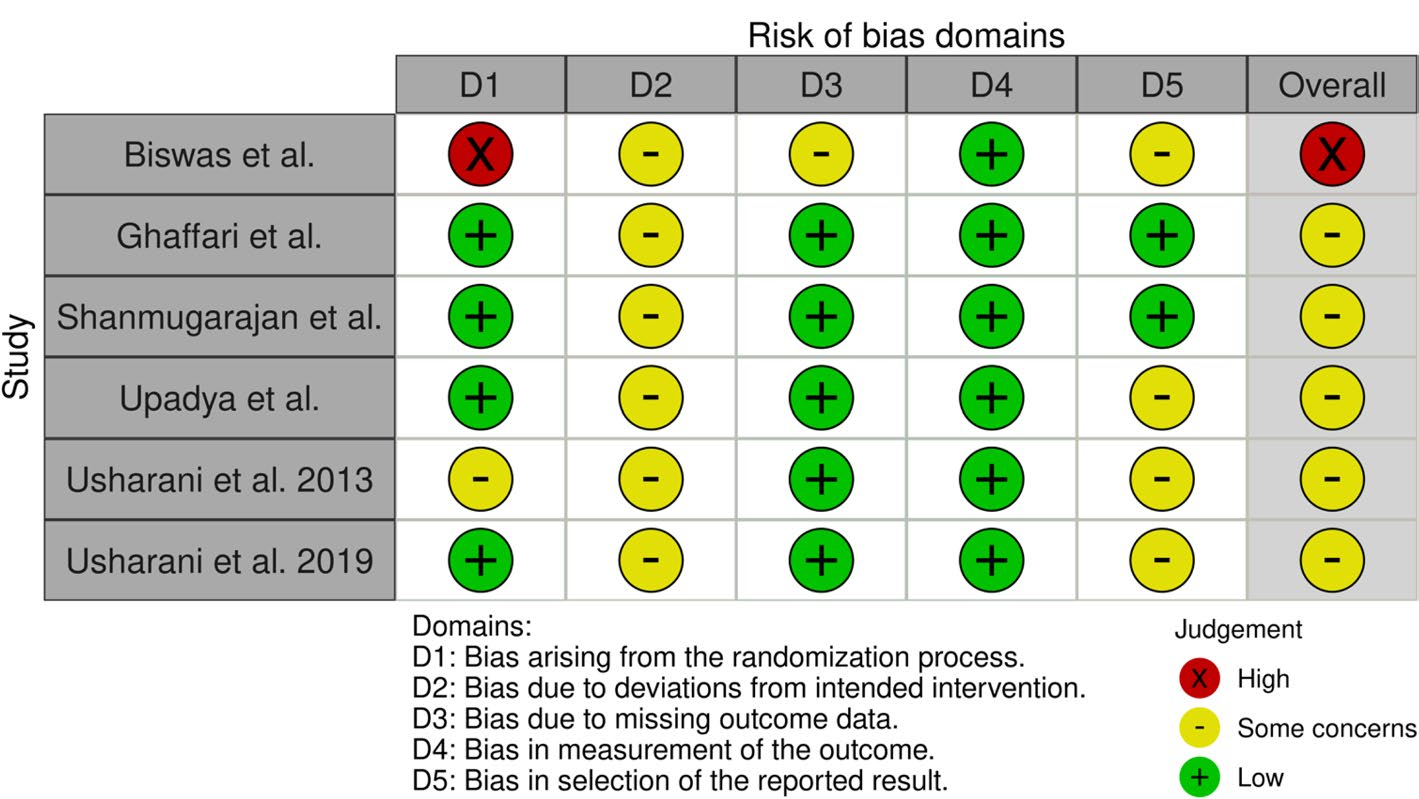
Cochrane Risk of Bias Assessments for parallel RCTS. Figure is generated using robvis [73]

**Fig. 3:**
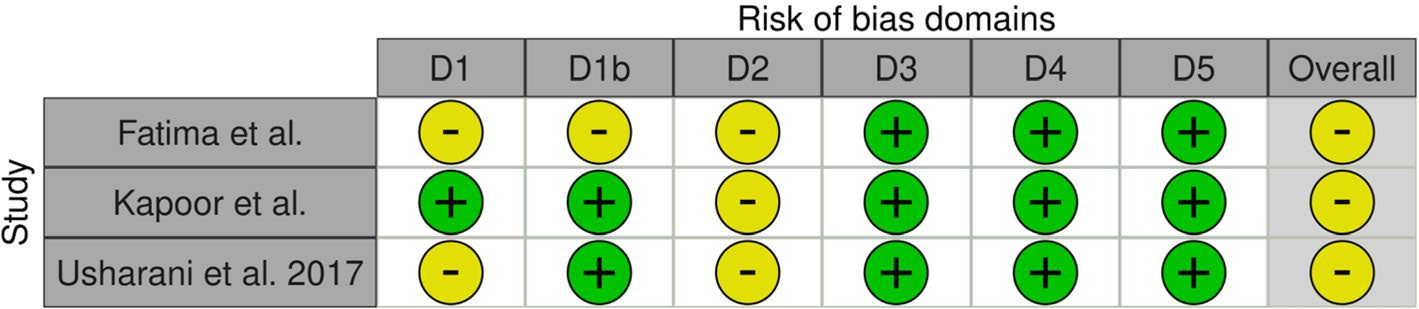
Cochrane Risk of Bias Assessments for cross-over RCTS. Figure is generated using robvis [73]

The methodological quality of all 9 RCTs, including the cross-over studies, were assessed using the ROB2 tool [62]. Of the parallel RCTS, five studies [52, 54–57] had an overall risk of some concern, and one [50] had a high risk of bias arising from the randomization process. All the cross-over studies [51, 53, 58] had an overall risk of some concern. Figures 2 and 3 show a detailed account of the risk of bias across each domain for the parallel and cross-over studies, respectively. All three cross-over studies [51, 53, 58] showed appropriate cross-over design. Two studies [53, 58] showed proper randomization order, while one study [51] was unclear because they did not describe the randomization method. None of the three cross-over studies explicitly discussed if there were any carry-over effects.

In terms of study outcomes, seven RCTs measured serum TC, TG, HDL-C, and LDL-C [50, 52–57]; four investigated serum VLDL-C [50, 54–56]; five examined systolic and diastolic blood pressure [51–54, 58]; and five reported serum hsCRP [50, 54–57].

Eight RCTs treated participants with an aqueous EO fruit extract [50, 51, 53–58] and the remaining RCT used powdered EO fruit [52]. *Emblica officinalis* was taken orally in capsule form for all nine RCTs. Dosage was 500 mg/day [50, 51, 53, 56, 57], 1000 mg/day [54–58], and 1500 mg/day [52]. *Emblica officinalis* fruit consumption was well-tolerated, with no included RCT reporting any adverse event serious enough to result in premature discontinuation of the study.

### Meta-analyses: Effect size by risk factor

**Fig. 4:**
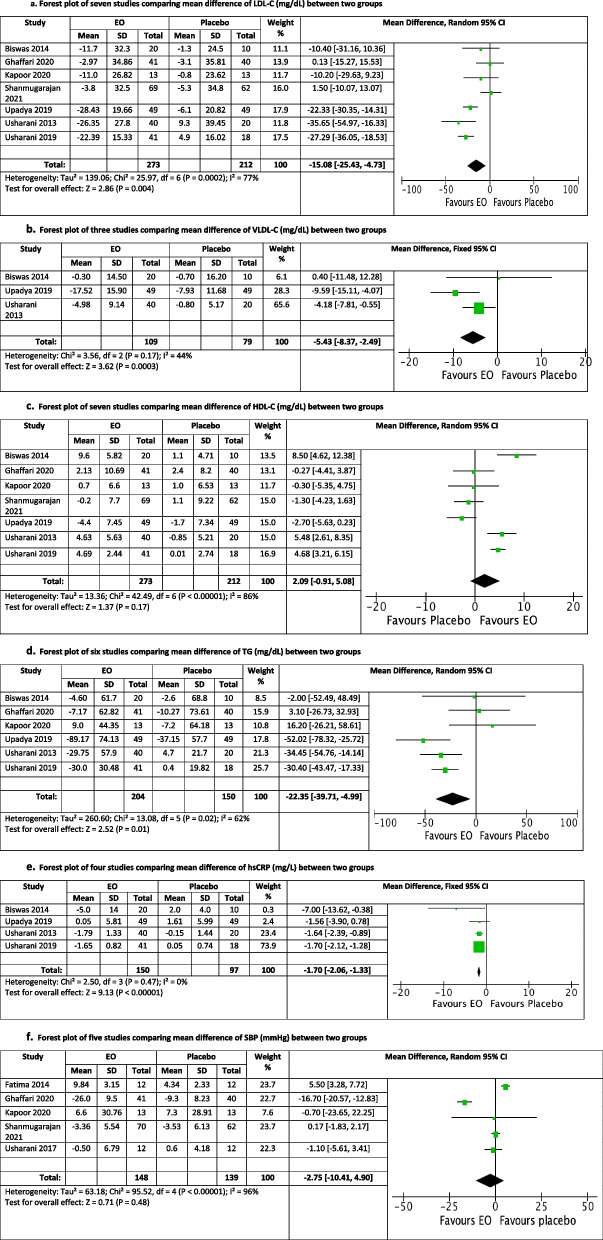
**a** Forest plot of seven studies comparing mean difference of LDL-C (mg/dL) between two groups. **b** Forest plot of three studies comparing mean difference of VLDL-C (mg/dL) between two groups. **c** Forest plot of seven studies comparing mean difference of HDL-C (mg/dL) between two groups. **d** Forest plot of six studies comparing mean difference of TG (mg/dL) between two groups. **e** Forest plot of four studies comparing mean difference of hsCRP (mg/L) between two groups. **f** Forest plot of five studies comparing mean difference of SBP (mmHg) between two groups. **g** Forest plot of five studies comparing mean difference of DBP (mmHg) between two groups. *EO Emblica officinalis*, *LDL-C* Low-density lipoprotein cholesterol, *VLDL-C* Very low-density lipoprotein cholesterol, *HDL-C* High-density lipoprotein cholesterol, *TG* Triglycerides, *hsCRP* High-sensitivity C-reactive protein, *SBP* Systolic blood pressure, *DBP* Diastolic blood pressure

The effects of EO ingestion on various CVD risk factors compared with placebo are presented in Fig. 4. *Emblica officinalis* ingestion had significant effects at lowering LDL-C (MD = -15.08 mg/dL [95% CI = -25.43 to -4.73]), I^2^ = 77%, prediction interval = -48.29 to 18.13, *p* = 0.004), VLDL-C (MD = -5.43 mg/dL [95% CI = -8.37 to -2.94], I^2^ = 44%, *p* = 0.0003), TG (MD = -22.35 mg/dL [95% CI = -39.71 to -4.99], I^2^ = 62%, prediction interval = -73.47 to 28.77, *p* = 0.01), and hsCRP (MD = -1.70 mg/L [95% CI = -2.06 to -1.33], I^2^ = 0%, *p* = 0. 00001). EO did not have a significant effect on HDL-C (MD = 2.09 mg/dL [95% CL = -0.91 to 5.08], I^2^ = 86%, prediction interval = -8.09 to 12.27, *p* = 0.17), SPB (MD = -2.75 mmHg [95% CL = -10.41 to 4.90], I^2^ = 96%, prediction interval = -30.93 to 25.43, *p* = 0.48) and DPB (MD = -0.83 mmHg [95% CL = -5.87 to 4.21], I^2^ = 89%, prediction interval = -19.09 to 17.43, *p* = 0.75). Many of the results show high statistical heterogeneity, where an I^2^ of 75% to 100% is reported as considerable heterogeneity [74]. The prediction intervals show there is a substantial range of effect size.

## Discussion

This review estimated the effect of EO consumption on physiological CVD risk factors. *Emblica officinalis* consumption showed statistically significant improvements in LDL-C, VLDL-C, HDL-C, TG, and hsCRP compared with placebo.

### Limitations

Considerable heterogeneity exists in the RCTs examining the effect of EO extract on CVD risk factors that have been published so far. There are marked variations in the participant inclusion criteria, baseline biochemical values, study design, and duration of treatment. The potential variation in proprietary extract preparation techniques between studies may have also influenced the findings. One RCT [52] did not use an extract but the dried raw EO fruit itself, which may exert a different effect at equal dosage relative to an extract. Commercial interest may limit the submission and subsequent publication of non-significant or opposing data regarding the alleged health benefits of EO. Relatively small sample sizes must also be taken into consideration, with three of the nine included RCTs containing only 12 [51, 58] or 13 [53] participants. There is also a limited number of included RCTs and neither funnel plots nor Eggar regression tests were completed due to insufficient sample size. Prediction intervals, which signify an absolute measure of heterogeneity, were provided for all random effects models to aid in the interpretation of the heterogeneity [68]. There is a limitation in the analysis of the cross-over trials given the limited availability of reported data. The analysis of cross-over trials as parallel trials does give rise to a unit-of-analysis error [65]. However, this analysis is conservative, and consequently the cross-over studies are underweighted [65]. According to the Friedewald equation, LDL-C is calculated as TC minus HDL-C minus VLDL-C [75]. Clinically, VLDL is often estimated as TG divided by 2.2 (if values are in mmol/L) or 5 (if values are in mg/dL) [75]. Although a higher serum HDL-C is considered protective against CVD, a higher HDL-C would contribute to a higher serum TC based on this equation, which is considered a risk factor for CVD. Therefore, the individual components of TC (e.g., LDL-C, HDL-C, VLDL-C, TG) may be more informative when assessing CVD risk compared with TC alone. Therefore, TC was not included in this review. Excluding non-English articles and not investigating safety in this review are additional limitations.

Dyslipidemia is a primary causal factor for the development of atherosclerosis and CVD [1]. Dyslipidemia refers to abnormally high serum TC (≥ 5.2 mmol/L or ≥ 200 mg/dL), LDL-C (≥ 3.4 mmol/L or ≥ 130 mg/dL), TG (≥ 1.7 mmol/L or ≥ 150 mg/dL), or low serum HDL-C (< 1.0 mmol/L or < 40 mg/dL) [1]. For every 1.0 mmol/L (38.67 mg/dL) reduction in LDL-C there is a 20–22% relative risk reduction for the development of CVD [76]. High-density lipoprotein cholesterol is thought to counteract the atherosclerotic process by inhibiting the oxidization of LDL-C and removing cholesterol from foam cells (i.e., lipid-laden macrophages within the arterial tunica intima) for transportation back to the liver [50]. Non-HDL-C includes chylomicron remnants, VLDL-C, intermediate-density lipoprotein cholesterol, lipoprotein(a), and LDL-C [76]. The components of non-HDL-C are atherogenic, apolipoprotein B-100 (Apo B) containing lipoproteins [76]. *Emblica officinalis* showed improvements in components of non-HDL-C in this review.

The mechanisms of how EO may exert its beneficial effects on lipid profile are not fully elucidated. Proposed mechanisms include interference of cholesterol absorption [77]; inhibition of hepatic 3-hydroxy-3-methylglutaryl coenzyme-A (HMG-CoA) reductase activity, resulting in decreased cholesterol synthesis [78]; and increase in lecithin‑cholesterol acyltransferase (LCAT) activity, resulting in greater cholesterol transfer to HDL for transport to liver for hepatic degradation and biliary excretion [78]. Increased transfer of cholesterol to HDL from other sources (e.g., LDL) via upregulation of lipoprotein transfer enzymes and/or proteins may partially explain the tendency for EO to increase HDL-C with a concurrent decrease in non-HDL-C components [33]. *Emblica officinalis* may be an addition to established dietary interventions to combat dyslipidemia such as the Portfolio diet, or adjunct to standard pharmacotherapy such as HMG-CoA reductase inhibitors.

HMG-CoA reductase inhibitors, also known as statins, are a class of medication widely prescribed to treat dyslipidemia, especially to lower serum LDL-C [1]. Statins lower cholesterol via inhibition of hepatic HMG-CoA reductase, the rate-limiting enzyme of cholesterol biosynthesis [76]. Reported side effects of statin use include myopathy, hepatotoxicity, and cephalgia [56]. Several clinical trials have compared the effect of EO and statins on blood lipids [35, 36, 56]. These trials reported similar improvements in lipids after a 500 – 1000 mg/day dose of EO and an initial dose (10 – 20 mg/day) of standard statin pharmacotherapy. However, only Usharani and colleagues [56] met inclusion criteria for this review (Table 1). No serious adverse events were reported in the EO or statin groups for the duration of these clinical trials.

C-reactive protein (CRP) is a non-specific biomarker of inflammation. Elevated hsCRP (≥ 2 mg/L) has been associated with atherosclerosis and CVD [1, 76]. However, it remains uncertain whether CRP is directly involved in the progression of atherosclerosis or simply a consequence of the atherosclerotic process [56]. Oxidative stress via accumulation of reactive oxygen species (ROS) may reduce the bioavailability of nitric oxide and result in endothelial dysfunction and vascular inflammation. Excess ROS may also increase the conversion of LDL-C to oxidized LDL-C, further exacerbating the inflammatory cascade [57]. The anti-inflammatory properties of EO—as demonstrated by the significant reduction in hsCRP in this meta-analysis—may be explained by the large antioxidant capacity and ROS scavenging ability of the fruit [56]. This antioxidant capacity is partially attributed to the relatively high ascorbic acid and ellagitannin content [53]. The EO fruit is rich source of ellagitannins—such as chebulagic acid, pedunculagin, geraniin, corilagin, elaeocarpusin—which are hydrolysable to ellagic acid and gallic acid [53]. The complex and potentially synergistic interactions between the various EO phytochemicals may also enhance the antioxidant capacity of the fruit [53].

## Conclusions

*Emblica officinalis* has beneficial effects on LDL-C, VLDL-C, HDL-C, TG, and hsCRP that are statistically significant; however, due to small sample size and heterogeneity (clinical and statistical), these results should be interpreted with caution. Further research on the clinical effects of EO is necessary. Additional large RCTs are required to confirm these results and identify the most efficacious dose and form of EO in various patient populations. Potential sex differences should also be explored. The mechanism of action requires further investigation as the many bioactive phytochemicals of the fruit appear to exert individual beneficial effects and the potential to interact synergistically. These complex, potentially synergistic interactions may favour consumption of the whole EO fruit as opposed to proprietary extracts of the fruit, where some of the bioactive phytochemicals may be lost or altered during the extraction process. However, this is speculatory and requires clinical validation involving minimally processed preparations of EO that can still be effectively blinded and placebo controlled. For example, dried EO fruit powder ingested via capsule. *Emblica officinalis* may offer an efficacious, affordable, and convenient option for primary or secondary prevention of CVD as either monotherapy or adjunct to evidence-based dietary patterns and/or standard pharmacotherapy.

## Supplementary Information

**Additional file 1. **Appendix A and B.

## Data Availability

All data supporting this systematic review and meta-analysis are from published randomized controlled trials that have been cited in this article.

## Abbreviations

Apo B: Apolipoprotein B-100
CRP: C-reactive protein
CVD: Cardiovascular disease
DASH: Dietary Approaches to Stop Hypertension
EO: *Emblica officinalis*
HDL-C: High-density lipoprotein cholesterol
HMG-CoA: Hepatic 3-hydroxy-3-methylglutaryl coenzyme-A
hsCRP: High-sensitivity C-reactive protein
LCAT: Lecithin‑cholesterol acyltransferase
LDL-C: Low-density lipoprotein cholesterol
MD: Mean difference
PRISMA: Preferred Reporting Items for Systematic Reviews and Meta-Analyses
RCTs: Randomized controlled trials
ROB2: Risk of Bias 2 Assessment Tool
ROS: Reactive oxygen species
T2DM: Type 2 diabetes mellitus
TC: Total cholesterol
TG: Triglycerides
VLDL-C: Very low-density lipoprotein cholesterol
