# Supplementary material for: Clinical effects of Emblica officinalis fruit consumption on cardiovascular disease risk factors: a systematic review and meta-analysis
Source: BMC Complement Med Ther. 2023 Jun 9;23:190. doi: 10.1186/s12906-023-03997-8 (PMC10251691; doi:10.1186/s12906-023-03997-8)

**Appendix A: Search Strategy Example**

**Web of Science**

TOPIC: (("Aamalaki" OR "Amalaki" OR "Amla" OR "Amloki" OR "Aonla" OR "Dhatriphala" OR "Emblic" OR "Emblica" OR "Groseillier de Ceylan" OR "Indian-Gooseberry" OR "Indian gooseberry" OR "Malacca Tree" OR "Mirobalano" OR "Mirobalanus embilica" OR "Myrobalan" OR "Neli" OR "Yu Gan Zi") AND ("Apolipoprotein*" OR "Arteriosclero*" OR "Atherogen*" OR "Atherosclero*" OR "Cardiovascular disease*" OR "Cerebrovascular disease*" OR "Cholesterol" OR "Clotting" OR "Coronary artery disease*" OR "Dyslipid*" OR "Endotheli*" OR "HDL" OR "Heart disease*" OR "Hypercholesterol*" OR "Hyperlipid*" OR "LDL" OR "Lipid*" OR "Lipoprotein*" OR "Metabolic syndrome*" OR "Peripheral artery disease*" OR "Platelet aggregation" OR "Stroke*" OR "Triacylglyceride*" OR "Triacylglycerol*" OR "Triglyceride*" OR "VLDL" OR "Blood glucose" OR "Blood sugar" OR "Diabet*" OR "Glycated hemoglobin" OR "Glycated haemoglobin" OR "Glycosylated hemoglobin" OR "Glycosylated haemoglobin" OR "Haemoglobin A1c" OR "Hemoglobin A1c" OR "Hyperglyc*" OR "HbA1c" OR "Prediabet*" OR "Arrhythmia*" OR "Arterial stiffness" OR "Blood pressure" OR "Cardioprotecti*" OR "Cardiotoxic*" OR "Heart failure" OR "Heart rate" OR "Hypertensi*" OR "Myocardial dysfunction*" OR "Vascular function*" OR "Antioxidant*" OR "C-reactive protein" OR "CRP" OR "hsCRP" OR "Inflammat*" OR "Oxidative stress*") )

Indexes=SCI-EXPANDED, SSCI, A&HCI, CPCI-S, CPCI-SSH, ESCI Timespan=All years

**Appendix B: Formula for combining intervention groups as suggested in the Cochrane handbook [66]**


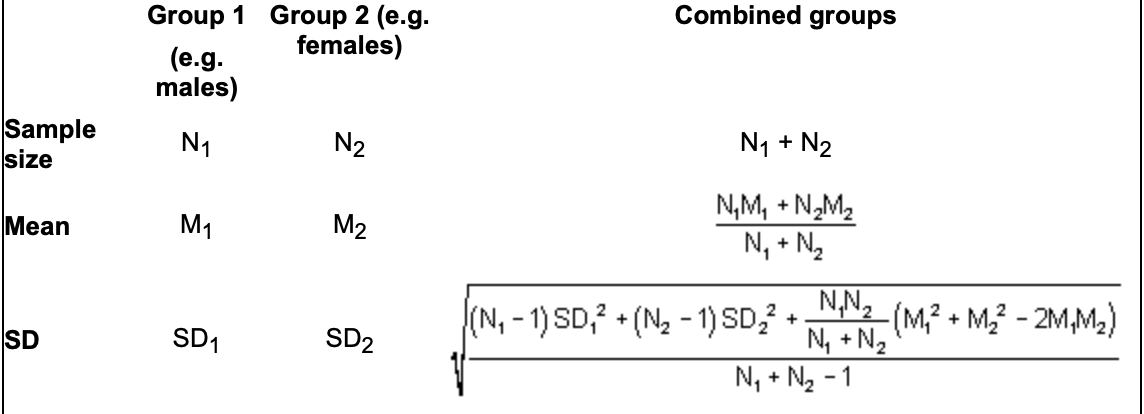

Supplement: Supplementary file 1 — Additional file 1. Appendix A and B. [file 12906_2023_3997_MOESM1_ESM.docx]
